# Challenges and facilitators for meaningful and impactful stakeholder engagement in global health research

**DOI:** 10.7189/jogh.16.03016

**Published:** 2026-07-24

**Authors:** Qorinah Estiningtyas Sakilah Adnani, Genevie Fernandes, Emma Kinley, Siân Williams

**Affiliations:** 1 Department of Public Health, Faculty of Medicine, Universitas Padjadjaran, Bandung, Indonesia; 2 International Primary Care Respiratory Group, Edinburgh, Scotland, UK; 3 Liverpool John Moores University, Liverpool, England, UK; 4 Usher Institute, University of Edinburgh, Edinburgh, Scotland, UK

**Keywords:** stakeholder engagement, respiratory health, global health, research, ethics, low- and middle-income country, LMIC

## Abstract

Stakeholder engagement is increasingly required in global health research, yet its implementation often remains underexamined, particularly in low- and middle-income countries. Drawing on literature and experience from a multinational respiratory research programme, this viewpoint highlights how structural constraints, ethical ambiguity, power dynamics, and socio-cultural factors can undermine meaningful engagement. Without reflexive, adequately resourced, and institutionally supported approaches, stakeholder engagement risks becoming symbolic rather than transformative. Funders and institutions must therefore invest in flexible, long-term, and context-sensitive engagement models that genuinely reflect and respond to the communities global health research seeks to serve.

Global health funders and policymakers increasingly expect researchers to demonstrate how research has been shaped by stakeholder engagement, guided by principles such as ‘no decision about me without me’ [1] and ‘leave no one behind’ [2]. However, much of the existing literature focuses on normative frameworks or practical guidance for engagement, with limited critical reflection on the unintended consequences, biases, and tensions that may arise when engagement is implemented in complex global health contexts.

This viewpoint contributes by offering a critical, reflexive lens on how and under what conditions stakeholder engagement becomes meaningful or risks reinforcing existing inequities, particularly in low- and middle-income countries (LMICs). Drawing on published literature and our collective experience within a global respiratory research programme (*i.e.* the Global Health Research Unit on Respiratory Health (RESPIRE)), we do not aim to provide a step-by-step implementation guide. Instead, we want to support researchers, institutions, and funders in understanding the implications of engagement-related design choices across the research lifecycle (**Table 1**).

**Table 1:** Conceptual framework: challenges and facilitators of stakeholder engagement

Type of challenge	Level	Illustrative example	Relevant facilitator
Structural	Policy	Limited pre-award funding; rigid funding timelines that do not allow early or sustained engagement	Flexible funding schemes; dedicated pre-award engagement funding; funder requirements that value engagement quality
Interpersonal	Project	Dominance of specific actors (*e.g.* senior researchers or external experts); unequal participation in decision-making; limited space for dissenting voices	Inclusive facilitation; shared decision-making processes; reflexive leadership that actively redistributes power
Ethical	Institutional	Unclear ethics requirements for engagement activities; limited guidance on compensation, consent, and roles of community partners	Ethics guidance and training. Clear ethics guidance for engagement; training on participatory ethics; institutional recognition of community partners as co-producers of knowledge
Sociocultural	Community	Gender norms and hierarchy, or social exclusion limiting participation of certain groups; language and cultural barriers	Culturally sensitive and gender-responsive approaches; use of trusted local facilitators; adaptation of engagement methods to local contexts

## DEFINING STAKEHOLDER ENGAGEMENT

Stakeholders include individuals or groups responsible for or affected by health- and healthcare-related decisions that research can inform, such as patients, local communities, health care providers, policymakers, and non-governmental organisations. Engaging stakeholders improves alignment of research with local priorities, supports implementation by fostering commitment to research findings and translating insights into operational detail, and promotes the uptake of findings in real-world settings [3]. Integrating stakeholders’ perspectives, values, and lived experiences not only improves ethical rigour, but also enhances the feasibility and contextual relevance of the evidence-based solutions [4]. This approach enriches our understanding of health issues and services, while also highlighting the real challenges faced by the affected communities [5]. While stakeholder engagement practices are increasingly recognised by funders as essential to impactful global health research [3,5], robust reporting and critical examination of these practices in global health research programmes, especially in LMICs, remains limited.

To anticipate the discussion that follows, challenges to stakeholder engagement in global health research can be broadly grouped into the following themes:

1. structural constraints, particularly funding and time;
2. communication and accessibility barriers;
3. ethical ambiguity and blurred roles;
4. power dynamics and representation;
5. socioeconomic, cultural, and political barriers.

## KEY CHALLENGES

### Funding constraints

Early engagement of stakeholders, such as during priority setting and grant development, is widely recognised as good practice in global health research. However, such engagement is rarely supported by dedicated pre-award funding, particularly in LMICs. Analyses of patient and public involvement (PPI) funding mechanisms indicate that engagement budgets are often released only after contracts are finalised, leaving early-stage activities dependent on institutional goodwill or personal commitments by local partners [3,5].

Experience from the RESPIRE programme suggests that pre-award engagement is, therefore, frequently informal and undocumented, which may unintentionally reinforce ‘convenience partnerships’ with already well-connected stakeholders. Similar dynamics have been described in global health collaborations, where limited early-stage funding can narrow representation and reproduce existing power asymmetries between institutions and actors [6]. Sustaining engagement beyond the funding period presents an additional challenge when formal resources and accountability mechanisms come to an end.

### Communication barriers

Stakeholders involved in global health research vary widely in language, literacy, health system experience, and access to digital technologies. Both face-to-face and digital engagement modalities present distinct accessibility challenges. In-person meetings may be constrained by travel costs, time, and geographic distance, while virtual engagement relies on digital literacy, stable internet access, and access to appropriate devices [6,7]. Beyond digital access, limited health literacy, such as low awareness and understanding of the health condition among caregivers and communities, can further constrain meaningful engagement, affecting stakeholders’ ability to interpret research aims, interventions, and outcomes.

Across RESPIRE partner countries, language barriers and limited digital access in remote or underserved areas frequently constrained participation by the most marginalised groups. These observations are presented as practice-based lessons and align with broader literature on the digital divide in LMICs and concerns about digital exclusion in participatory research [8].

### Ethical ambiguity

Is ethics approval required for stakeholder engagement? Uncertainty regarding the requirement for formal ethics approval for stakeholder engagement remains common in global health research. While some jurisdictions exempt PPI activities from formal ethics review, others require approval, which may delay engagement or create confusion among research teams [8–10]. In practice, researchers often err on the side of caution by seeking ethical approval, even where formal review may not be strictly required. Ethical concerns are particularly salient when clinicians engage their own patients as stakeholders. Participatory research literature highlights risks related to perceived coercion, blurred professional boundaries, and therapeutic misconception in such contexts [10,11].

### What’s in it for us?

While global health researchers often benefit from stakeholder engagement through improved recruitment, implementation, and contextual understanding [9], immediate benefits for community stakeholders are less evident and depend on the available resources and prevailing norms. Long-term benefits, such as improved access to health care services, take time and require health system capacity. In communities with weak health systems and historical mistrust of healthcare institutions, a lack of immediate and tangible benefits can limit interest and participation.

### Power dynamics

Power imbalances permeate global health research: between institutions in high-income countries and LMICs, between researchers and communities, and within countries across stakeholders [12]. Sociocultural norms influence which stakeholder is legitimate, which, in turn, can enable them to have a louder voice and power over agendas, priorities, and decisions. In South Asia, for instance, power asymmetry exists between clinicians in primary and secondary care and between private and public systems, as well as between patients and clinicians, which can curtail the expression of lived experiences and challenges with the health service. High-power distance cultures make open dialogue and shared decision-making difficult, especially for junior researchers tasked with managing these relationships [13]. The concept of high-power distance refers to cultural contexts in which hierarchical relationships are widely accepted and rarely challenged, shaping patterns of authority, voice, and decision-making [14]. Early-career researchers often face additional challenges due to their position within academic and social hierarchies, particularly when tasked with facilitating inclusive dialogue in culturally stratified environments [6].

### Socioeconomic, cultural, and political barriers

Lost wages, travel costs, household responsibilities, gender norms, and requirements for family or community consent may limit stakeholder participation. Evidence from fragile and conflict-affected settings further indicates that political instability and crises, such as elections or conflict, can significantly disrupt planned engagement processes [15].

## FACILITATORS

### Institutionalisation of stakeholder engagement

Embedding stakeholder engagement within institutional policies and systems is foundational. This includes dedicated budget lines, clear ethical guidance, recognition within academic reward structures, and designated units or roles to support engagement across projects. Mitigation strategies include clear separation of clinical and research roles, third-party facilitation of informed consent processes, transparency regarding the purpose and limits of engagement, and institutional oversight supported by ongoing ethics training. Leadership commitment and feedback mechanisms are essential for sustaining practice [16].

**Figure 1:**
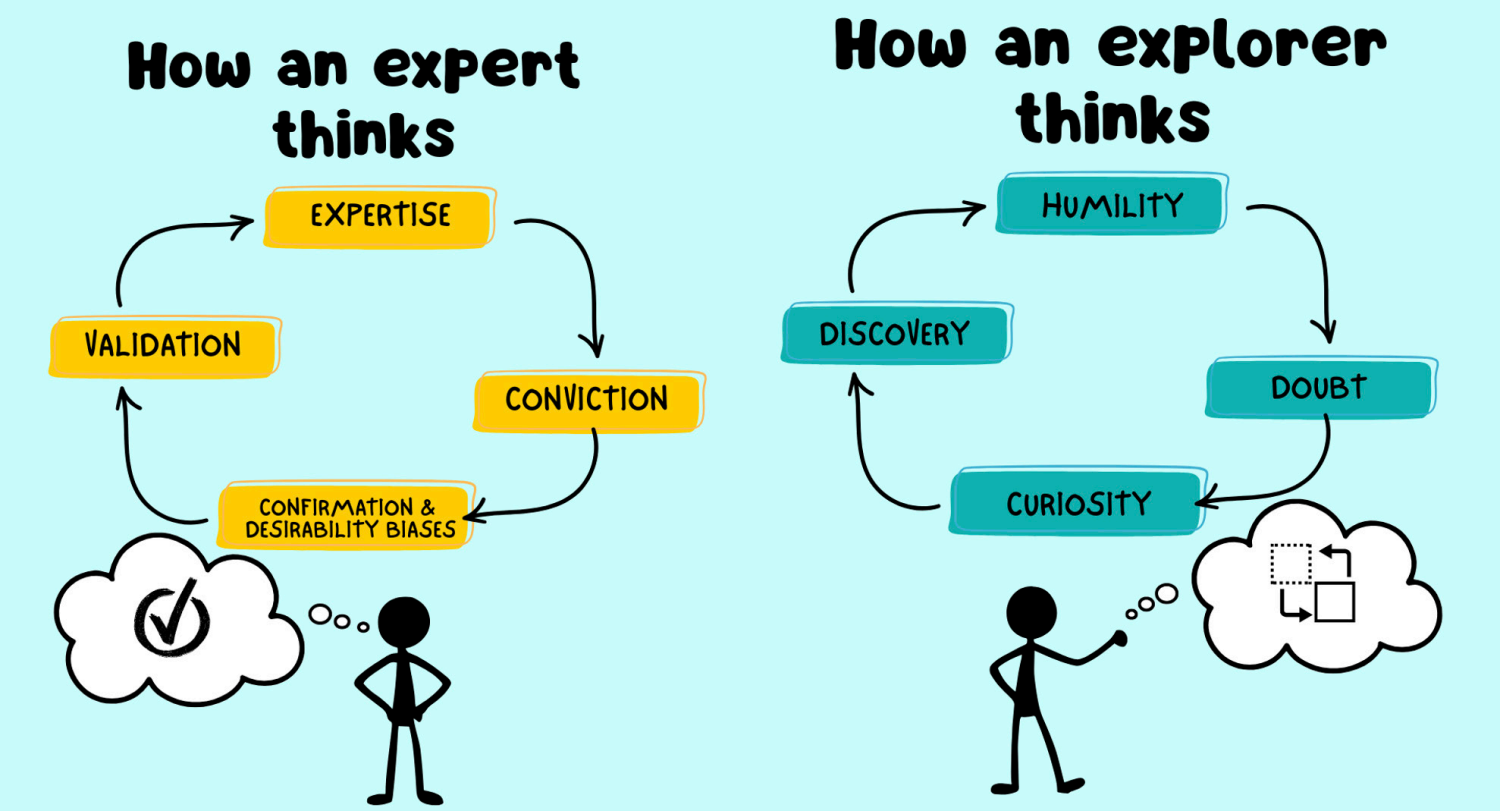
How and expert thinks *vs*. how an explorer thinks. Source: Helen Bevan, 2025 [17]. Adapted from a graphic by Anuj Magazine based on the book ‘Think Again’ by Adam Grant. Used with permission.

Further, if the goal of the research, which is often the case in global health research, is change for improvement, then we must integrate research behaviours that enable change – such as embedding a culture of humility, doubt, curiosity and discovery, rather than validating expertise (**Figure 1**) [17].

### Early and consistent stakeholder engagement

Involving stakeholders upstream in the priority-setting phase brings the research closer to the needs and priorities of its stakeholders, particularly the affected local communities. Early engagement indicates humility on the part of the researchers to listen to, learn from, and integrate stakeholders’ experiences, challenges, and perspectives into the study design; it also fosters trust and mutual learning, enables course corrections, and influences research adoption [18]. However, this requires funder flexibility, including grant review criteria that value adaptation based on stakeholder input rather than rigid adherence to pre-specified protocols.

### Relationship-building

Transparent and respectful communication with stakeholders, even during periods without major updates or when faced with disagreements, builds enduring relationships. Leveraging existing connections with stakeholders can help to establish initial relationships, but sustaining them requires continuous and deliberate effort. Recognising stakeholder contributions through authorship or acknowledgement in publications is one way to demonstrate respect and foster trust [19].

### Evaluation and learning

A culture of systematic evaluation is essential for assessing both the short- and long-term impacts of stakeholder engagement. Frameworks for evaluating PPI emphasise that meaningful indicators should capture influence, process quality, and sustainability, rather than relying solely on activity counts [3]. Illustrative, low-burden metrics include the proportion of protocol modifications informed by stakeholder input, stakeholder perceptions of influence on decision-making, and continuity of engagement beyond the funding period. Such indicators can be integrated into routine project monitoring and learning systems without imposing excessive demands on research teams.

### Creativity and adaptability

The COVID-19 pandemic compelled global health researchers to innovate and adapt to sustain stakeholder engagement [10]. These methods remain relevant beyond crises. However, researchers still confront common challenges such as budget constraints, difficulties in capturing stakeholders’ attention due to competing priorities, or the inability to reach vulnerable groups. Research teams need support to explore other avenues of resource mobilisation, including community spaces and events, virtual events, or harmonising funding from aligned partners. Creative methods, including arts and crafts, storytelling, street plays, as well as virtual engagement activities, can be appealing and accessible to stakeholders, encouraging greater participation and collaboration. The downscaling of foreign aid spending on global health research projects puts such approaches under threat unless bolstered by local investment or philanthropic support [20].

## CONCLUSIONS

Stakeholder engagement is not an ancillary activity, but a core component of equitable and impactful global health research. Without structural support, ethical clarity, and ongoing evaluation, engagement risks becoming symbolic or reinforcing existing inequities and biases particularly those related to power imbalances, whose knowledge is valued, and who ultimately benefits from research processes and outcomes. Approaches grounded in reflexivity and humility are therefore essential to challenge these dynamics rather than reproduce them. Funders should support longer-term and more flexible engagement models, including pre-award funding. Institutions must invest in systems and capacities that sustain meaningful engagement throughout and beyond the research lifecycle. Only then can global health research more authentically reflect the communities it seeks to serve.

## References

[1] Delbanco T Berwick DM Boufford JI Edgman-Levitan S Ollenschläger G Plamping D Healthcare in a land called PeoplePower: nothing about me without me. Health Expect. 2001;4:144–50. 10.1046/j.1369-6513.2001.00145.x 11493320 PMC5060064

[2] Nelson E, Apolot RR, Rasheed S, Contractor S, Flores W. What does it mean to take a 'leave no one behind' approach to community engagement and involvement in global health research? 14 September 2021. Available: https://www.nihr.ac.uk/what-does-it-mean-take-leave-no-one-behind-approach-community-engagement-and-involvement-global-health-research. Accessed: 24 January 2026.

[3] Boaz A Hanney S Borst R O’Shea A Kok M How to engage stakeholders in research: design principles to support improvement. Health Res Policy Syst. 2018;16:60. 10.1186/s12961-018-0337-6 29996848 PMC6042393

[4] Bate P Robert G Experience-based design: from redesigning the system around the patient to co-designing services with the patient. Qual Saf Health Care. 2006;15:307–10. 10.1136/qshc.2005.016527 17074863 PMC2565809

[5] National Institute for Health and Care Research. Community engagement and involvement. Available: https://www.nihr.ac.uk/research-funding/global-health/community-engagement-and-involvement. Accessed: 24 January 2026.

[6] Abimbola S The uses of knowledge in global health. BMJ Glob Health. 2021;6:e005802. 10.1136/bmjgh-2021-005802 33820807 PMC8030475

[7] Tindana P de Vries J Campbell M Littler K Seeley J Marshall P Community engagement strategies for genomic studies in Africa: a review of the literature. BMC Med Ethics. 2015;16:24. 10.1186/s12910-015-0014-z 25889051 PMC4407431

[8] Suri S Harrison SL Bevin-Nicholls A Shenton F Atkinson S Earle J Patient and public involvement and engagement: Do we need an ‘ethical anchor’? Res Involv Engagem. 2024;10:113. 10.1186/s40900-024-00624-9 39482787 PMC11526663

[9] Adhikari B Pell C Cheah P Community engagement and ethical global health research. Glob Bioeth. 2019;31:1–12. 32002019 10.1080/11287462.2019.1703504 PMC6968663

[10] Fernandes G Jackson T Kashif A Rahman AE Roy AK Asmd AI Sustaining stakeholder engagement for health research during the COVID-19 pandemic: Lessons from the RESPIRE programme in Bangladesh, India, Malaysia, and Pakistan. J Glob Health. 2022;12:03057. 10.7189/jogh.12.03057 36056799 PMC9440618

[11] Council for International Organizations of Medical Sciences. International Ethical Guidelines for Health-related Research Involving Humans. 4th ed. Geneva, Switzerland: Council for International Organizations of Medical Sciences; 2016. Available: https://cioms.ch/wp-content/uploads/2017/01/WEB-CIOMS-EthicalGuidelines.pdf. Accessed: 24 January 2026. 40523065

[12] Khan M Abimbola S Aloudat T Capobianco E Hawkes S Rahman-Shepherd A Decolonising global health in 2021: a roadmap to move from rhetoric to reform. BMJ Glob Health. 2021;6:e005604. 10.1136/bmjgh-2021-005604 33758016 PMC7993212

[13] The Culture Factor. Country Comparison tool. Available: https://www.theculturefactor.com/country-comparison-tool?countries=indonesia. Accessed: 24 January 2026.

[14] Hofstede G, Hofstede GJMM. Cultures and organizations: software of the mind. I ntercultural Cooperation and Its Importance for Survival. 3rd ed. New York, USA: McGraw-Hill; 2011.

[15] Murphy J Qureshi O Endale T Esponda GM Pathare S Eaton J Barriers and drivers to stakeholder engagement in global mental health projects. Int J Ment Health Syst. 2021;15:30. 10.1186/s13033-021-00458-y 33812375 PMC8019163

[16] Uneke CJ Okedo-Alex IN Akamike IC Uneke BI Eze II Chukwu OE Institutional roles, structures, funding and research partnerships towards evidence-informed policy-making: a multisector survey among policy-makers in Nigeria. Health Res Policy Syst. 2023;21:36. 10.1186/s12961-023-00971-1 37237324 PMC10223846

[17] Bevan H. Helen Bevan graphics posted. 2025. Available: https://www.slideshare.net/slideshow/helen-bevan-graphics-posted-2025-2-pdf/284921779. Accessed: 10 January 2026.

[18] Rahman AE Jabeen S Fernandes G Banik G Islam J Ameen S Introducing pulse oximetry in routine IMCI services in Bangladesh: A context-driven approach to influence policy and programme through stakeholder engagement. J Glob Health. 2022;12:06001. 10.7189/jogh.12.06001 35441007 PMC8994831

[19] Lock Ngiyampaa MJ McMillan Wiradjuri F Warne Oglala Lakota D Bennett Gamilaraay B Kidd Ngāpuhi J Williams Bkejwanong N ICIRAS: Research and reconciliation with indigenous peoples in rural health journals. Aust J Rural Health. 2022;30:550–8. 10.1111/ajr.12905 35859346 PMC9543535

[20] Nilima Gulrajani JP. With the knives out on development spending, have we reached ‘peak aid’? The Guardian. 23 January 2025. Available: https://www.theguardian.com/global-development/2025/jan/23/global-development-economics-donor-spending-refugee-oecd-world-bank-peak-aid?CMP=share_btn_url. Accessed: 10 January 2026.

